# UVC, UVB and UVA susceptibility of Phi6 and its suitability as a SARS-CoV-2 surrogate

**DOI:** 10.3934/microbiol.2022020

**Published:** 2022-07-08

**Authors:** Laura Weyersberg, Eva Klemens, Jule Buehler, Petra Vatter, Martin Hessling

**Affiliations:** Ulm University of Applied Sciences, Department of Medical Engineering and Mechatronics, Albert Einstein-Allee 55, D-89081 Ulm, Germany

**Keywords:** SARS-CoV-2 surrogate, Phi6, ultraviolet radiation, radiation disinfection, UVA, UVB, UVC

## Abstract

For SARS-CoV-2 disinfection systems or applications that are based on UVC, UVB or UVA irradiation, it would be desirable to have a SARS-CoV-2 surrogate for tests and development, which does not require a laboratory with a high biosafety level. The bacteriophage Phi 6, an enveloped RNA virus like coronaviruses, is an obvious candidate for such a surrogate. In this study, UVC, UVB and UVA log-reduction doses for Phi6 are determined by plaque assay. Log-reduction doses for SARS-CoV-2 are retrieved from a literature research. Because of a high variability of the published results, median log-reduction doses are determined for defined spectral ranges and compared to Phi6 data in the same intervals. The measured Phi6 log-reduction doses for UVC (254 nm), UVB (311 nm) and UVA (365 nm) are 31.7, 980 and 14 684 mJ/cm^2^, respectively. The determined median log-reduction doses for SARS-CoV-2 are much lower, only about 1.7 mJ/cm^2^ within the spectral interval 251–270 nm. Therefore, Phi6 can be photoinactivated by all UV wavelengths but it is much less UV sensitive compared to SARS-CoV-2 in all UV spectral ranges. Thus, Phi6 is no convincing SARS-CoV-2 surrogate in UV applications.

## 1. Introduction

For the past two years, the world has been in the grip of the coronavirus pandemic caused by the SARS CoV-2 (coronavirus) with 500 million confirmed infections and 6.2 million recorded deaths to date [1]. SARS-CoV-2 is an enveloped single-stranded RNA virus, of zoonotic origin, which causes a respiratory disease known as COVID-19 (coronavirus disease 2019) [2],[3]. The virus is transmitted through air and is highly contagious. Coronavirus vaccines are meanwhile available, but despite approximately 10 billion doses of vaccine administered, the spread of the pandemic has not yet been halted [1]. Therefore, hygiene measures like the use of facemasks and the disinfection of surfaces or air are still mandatory. Fortunately, the coronavirus is sensitive to known chemical and physical disinfection methods, such as the application of chemical disinfectants, heat sterilization, or ultraviolet (UV) radiation [4]–[8].

However, there are processes and devices, such as air disinfection systems, where it is difficult to quantitatively assess the disinfection effect. Conducting experiments with coronaviruses requires a laboratory with biosafety level 3, which is often unavailable. Other human and animal coronaviruses such as HCoV OC43, HCoV 229E or BCoV are also pathogens but they are often employed as suitable surrogates because they are assumed to be very similar to SARS-CoV-2 and require only a biosafety level 2 laboratory [9]–[19].

Even more desirable, however, would be a SARS-CoV-2 surrogate that is nonpathogenic. Widespread bacteriophages as MS2, Q beta, PhiX174, T1, T4, T7, or Phi6 are obvious candidates, as they are not only nonpathogenic but also easy to handle. Among these phages Phi6 appears to be the most appropriate coronavirus surrogate, because like coronaviruses, Phi6 is an enveloped RNA virus, with the difference that its RNA is shorter and double-stranded [20],[21]. Additionally, in the past, the bacteriophage Phi6, has been successfully investigated or even successfully employed as a surrogate for coronaviruses in various applications [10],[22]–[32].

The study presented here will address the question of whether Phi6 is a suitable SARS-CoV-2 surrogate for virus inactivation by ultraviolet radiation. So far, three quantitative studies on the UVC (200–280 nm) sensitivity of Phi6 exist [29],[31],[33], but they differ from each other by up to a factor of 6. There is no available information of the sensitivity of Phi6 towards UVB (280–315 nm) or UVA (315–400 nm) irradiation, though these wavelengths are also known for their antimicrobial and antiviral features.

For SARS-CoV-2, there are several published UVC results available, but they differ even by a factor of about 500 between extremes, which hinders a meaningful comparison to the Phi6 properties. Additionally, some UVB and UVA SARS-CoV-2 data also exist, that could be compared to Phi6, if Phi6 results in these wavelength regions were available.

Therefore, in this study the UVC, UVB and UVA inactivation properties of Phi6 were investigated experimentally, and were compared to the results of a SARS-CoV-2 literature analysis to assess the suitability of Phi6 as a SARS-CoV-2 surrogate in potential UVC, UVB and UVA disinfection applications.

## 2. Materials and methods

### 2.1. Ultraviolet irradiation of Phi6 and plaque assay

Phi6 (DSM 21518) and its host *Pseudomonas syringae* (DSM 21482) were obtained from Deutsche Sammlung von Mikroorganismen und Zellkulturen GmbH (Braunschweig, Germany). *P. syringae* was propagated in tryptic soy broth (TSB, Sigma-Aldrich, St. Louis, USA). 25 mL of TSB were inoculated with a single *P. syringae* colony. The culture was grown overnight at 170 rpm and 25 °C in order to obtain an optical density of 0.20 to 0.25 at 600 nm, which was equal to 1–5 x 10^8^ colony-forming units (CFU)/mL.

**Figure 1.**
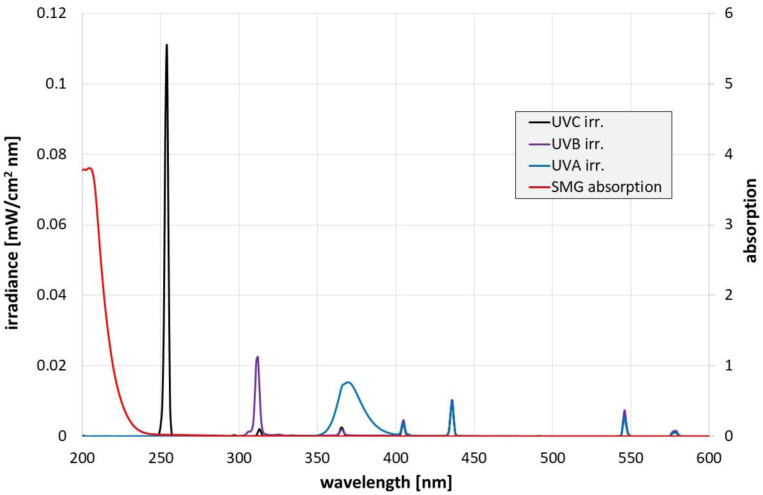
Measured spectral irradiances of the different UV lamps and SMG absorption.

A Phi6 stock solution of 10^9^ plaque-forming units (PFU)/mL of SMG buffer (saline magnesium gelatin buffer) was prepared as described by Sambrook and Russel [34]. For the irradiation experiments, the concentrated virus was diluted in SMG by a factor of 100 to 10^7^ PFU/mL and two 5 mL quartz beakers were filled with 2 mL each of this diluted virus sample solution. The filling height was just under 10 mm. The SMG absorption at 10 mm path length measured with a Specord Plus absorption spectrometer of Analytik Jena (Jena, Germany) is presented in Figure 1 and reveals no significant UV absorption above 240 nm.

The irradiation was performed with different ultraviolet bulbs in a distance of 32 cm above the sample: UVC (254 nm) bulb type Puritec HNS-S of Osram (Munich, Germany), UVB (311 nm) type UVB medical PLS and UVA (365 nm) PLS, both of Philips (Amsterdam, The Netherlands). The lamps were without reflectors or other optical elements. The quartz beaker containing the virus suspension was about 2 cm in diameter, which is small compared to the 32 cm distance, and thus the irradiation was assumed to be very homogeneous. The virus solution was not stirred during irradiation, however, it was mixed with the tip of the pipette just before sampling. The irradiation intensities were measured with a calibrated UV-VIS-spectroradiometer CAS 140 D of Instrument Systems (Munich, Germany).

As can be observed in Figure 1, the UV lamps also emit at longer wavelengths in the visible spectral range. However, since these emissions are weaker than the UV emissions and the antimicrobial properties of visible light are also weaker than those of UV radiation [35], these long-wavelength components were ignored.

Repeated temperature measurements with an infrared thermometer type Ranger MX4 of Raytek (Berlin, Germany) were also performed to detect a possible heating of the virus solution.

Post exposure, the Phi6 virus concentration was determined using the double agar overlay plaque assay as described by Kropinski et al. [36]: 100 µL of sequentially diluted Phi6 samples in SMG, 100 µL of host bacteria *P. syringae*, and 3 mL of soft TSB agar were mixed and plated over 90 mm TSB agar plates. Plaques were counted after 24 h of incubation at 25 °C and virus concentration was expressed in PFU/mL. At each sampling time point, three technical replicates were performed, and each irradiation wavelength was investigated in at least three independent experimental runs.

### 2.2. SARS-CoV-2 literature research and analysis

A literature search was performed in PubMed and Google Scholar using various combinations of the following terms: “SARS-CoV-2”, “disinfection”, “reduction”, “inactivation”, “UVC”, “UV-C”, “UVB”, “UV-B”, “UVA”, “UV-A”, “far-UVC”, “UV”, and “ultraviolet”. References in the retrieved literature were examined for their possible inclusion and references citing the identified literature were also checked.

For the analysis of the different UV sensitivities, the observed doses for a 3 log-reduction were retrieved from text, tables or figures and an average log-reduction dose (90% reduction) was calculated. Sample properties like the kind of liquid or surface were recorded. The results of SARS-CoV-2 irradiation in liquids were sorted by wavelengths and formally divided into spectral ranges of 20 nm width of 211–230 nm, 251–270 nm, 271–290 nm, 291–310 nm and 351–370 nm with the intention to get several results in each range. In the next step, median log-reduction doses were determined for each spectral range, as a high variability of the published single results-caused by different experimental setups and especially solutions with different UV absorption properties–was expected. The results of SARS-CoV-2 irradiation on surfaces were not included in the median determination, because the different surface samples of different materials and porosity are assumed to result in even larger variations as for the different liquid samples.

## 3. Results

### 3.1. Ultraviolet irradiation of Phi6 and plaque assay

**Figure 2.**
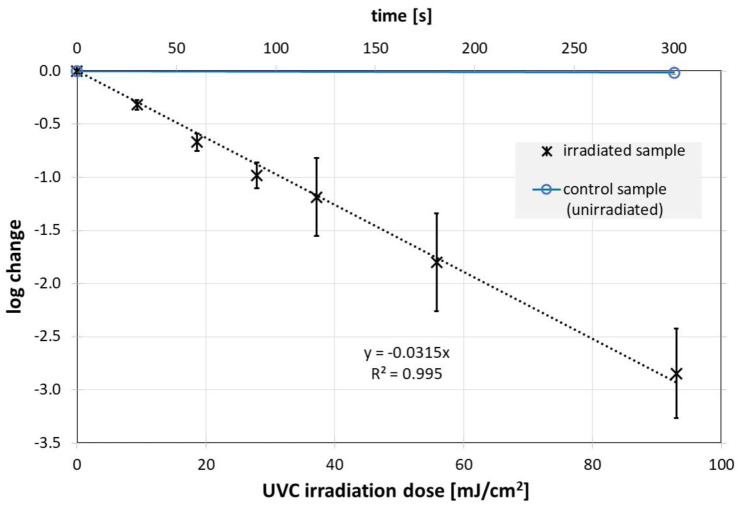
Inactivation of Phi6 as a function of the UVC (254 nm) irradiation. *Note: The phage titers in the irradiated (black) and non-irradiated samples (blue) were determined using the double agar overlay. Error bars represent the standard deviation of at least three independent experiments. Also given are the results (slope and coefficient of determination) of a linear regression.

**Figure 3.**
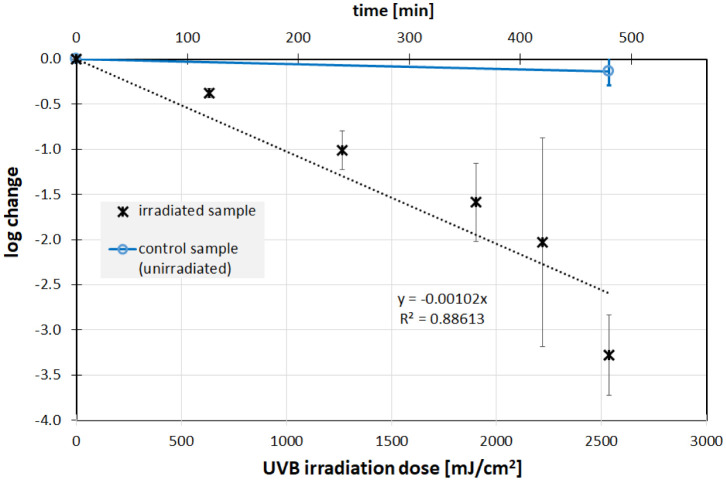
Inactivation of Phi6 as a function of the UVB (311 nm) irradiation. *Note: The phage titers in the irradiated (black) and non-irradiated samples (blue) were determined using the double agar overlay. Error bars represent the standard deviation of at least three independent experiments. Also given are the results (slope and coefficient of determination) of a linear regression.

**Figure 4.**
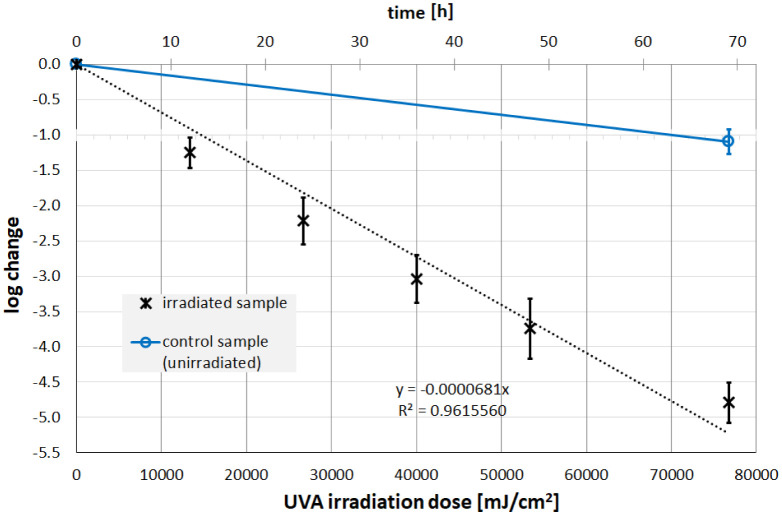
Inactivation of Phi6 as a function of the UVA (365 nm) irradiation. *Note: The phage titers in the irradiated (black) and non-irradiated samples (blue) were determined using the double agar overlay. Error bars represent the standard deviation of at least three independent experiments. Also given are the results (slope and coefficient of determination) of a linear regression.

The measured UV irradiation intensities for UVC (200–280 nm), UVB (280–315 nm) and UVA (315–400 nm) were 0.310, 0.088 and 0.309 mW/cm^2^, respectively. The irradiation times were chosen so that a reduction of about 99.9 % was expected. This meant approximately 300 s, 8 h and 69 h for UVC, UVB and UVA. The temperature in the individual samples fluctuated between 18 °C and 23 °C over the entire test period, so that temperature effects could be neglected. The results of the irradiation experiments describing the change in the Phi6 concentration are presented in Figures 2, 3 and 4.

The experiments reveal a successful Phi6 photoinactivation for all three ultraviolet wavelengths. The progression of virus concentration decrease appears to be approximately exponentially dependent on irradiance, i.e., linear in the semi-logarithmic plots in Figures 2, 3, and 4. The corresponding average log-reduction doses are 31.7, 980 and 14 684 mJ/cm^2^ for UVC, UVB and UVA, respectively.

### 3.2. SARS-CoV-2 literature research and analysis

During the literature search, about 25 publications on UV irradiation of SARS-CoV-2 could be retrieved. Most experiments were carried out with UVC radiation at a wavelength of 254 nm, the peak emission wavelength of low-pressure mercury vapor lamps, but some authors used UV-LEDs of different wavelengths. The SARS-CoV-2 samples were in UV absorbing and non-absorbing liquids or on various surfaces, resulting in different mean log-reduction doses given in Table 1. For the probably most important spectral interval [251–270 nm], which includes the peak emission wavelength of low-pressure mercury vapor lamps, the median log-reduction dose is only 1.7 mJ/cm^2^.

**Table 1.** Single SARS-CoV-2 log-reduction doses for different wavelengths in and on different media as retrieved from published literature and median SARS-CoV-2 log-reduction doses for selected spectral regions.

median log-reduction dose in mJ/cm^2^ [spectral range]	single result wavelength in nm	single log-reduction dose in mJ/cm^2^	Sample [ref.]
1.15 [211–230 nm]	222	0.7	PBS (liquid) [11]
	222	1.6	DMEM (liquid) [37]
	222	1.2	plastic (surface) [38]
	222	1.2	plastic (surface) [39]

1.7 [251–270 nm]	254	1.1	DMEM (liquid) [40]
	254	1.3	PBS (liquid) [11]
	254	1.5	PBS (liquid) [41]
	254	1.7	DMEM/PBS [19]
	254	2	DMEM? (liquid) [42]
	254	2.2	DMEM-HD (liquid) [43]
	254	2.7	DMEM (liquid) [44]
	254	5.3	DMEM (liquid) [45]
	254	5.3	DMEM (liquid) [46]
	254	*reduction observed but no dose available	airway epithelial cells ("liquid") [47]
	254	*< 0.6	plastic (surface) [48]
	254	*1.7 "wet" *1.3 "dry"	"wet" (liquid) "dry" (plastic surface) [49]
	254	*5.4 @ plastic *3.9 @ steel *<2.2 @ glas	plastic, steel, glass (surface) [50]
	254	*10 @ plastic *16 @ makeup *17 @ lipstick	plastic, powder and lipstick (surface) [51]
	254	*< 540	plastic (surface) [52]
	254	*reduction observed	different N95 respirators [53]
	254	*reduction observed but no dose available	plastic (surface) [54]
	265	1.5	PBS (liquid) [41]
	265	0.6	different media (liquid) [55]
	267	1.9	PBS (liquid) [9]
	270	1.1	PBS (liquid) [11]
	270	*4.4	glass (surface) [56]

2.3 [271–290 nm]	275	reduction observed but no dose available	unknown (liquid) [57]
	278	1	DMEM (liquid) [58]
	279	2.3	PBS (liquid) [9]
	280	2.8	PBS (liquid) [41]
	280	12.1	PBS (liquid) [59]
	280	1	different media (liquid) [55]
	282	1.9	PBS (liquid) [11]
	286	4.3	PBS (liquid) [9]

10.7 [291–310 nm]	297	10.7	PBS (liquid) [9]
	300	7.7	different media (liquid) [55]
	308	154	DMEM (liquid) [58]
2 569 [351–370 nm]	365	278	DMEM? (liquid) [42]
	366	4860	DMEM (liquid) [58]
	UVC & UVB & UVA (pulsed xenon light)	*reduction observed but no dose available	plastic and N95 respirators (surface) [60]
	UVB & UVA (simulated sun light)	*207 (UVB)	PBS (liquid) [61]
	UVB & UVA (simulated sunlight)	*33.6 (UVB) @ saliva *73.7 (UVB) @ GMEM	GMEM or simulated saliva on steel (surface) [62]

*Note: Results marked with “*” were not included in the determination of the median, because they were obtained on very different surfaces or information, e.g. irradiation dose was missing. (DMEM, EMEM, GMEM: different cell culture media with strong UV absorption, PBS: phosphate buffered saline with low UV absorption.)

## 4. Discussion and conclusions

All UV spectral ranges examined in the present work show antiviral properties towards Phi6, with UVC proving to be the most effective and UVA the least one. Table 2 lists a comparison of the results presented here with the radiation doses for a Phi6 log-reduction already described in the literature. The here observed Phi6 UVC log-reduction dose of 31.7 mJ/cm^2^ is more than twice as high as the value reported by Ye et al. [29] but in in excellent agreement with the results of Ma et al. [31]. If this study is included in the determination of the median, the Phi6 log-reduction dose is about 31.5 mJ/cm^2^.

**Table 2.** Single Phi6 log-reduction doses for different wavelengths in and on different media as retrieved from published literature and median Phi6 log-reduction doses for selected spectral regions.

median log-reduction dose in mJ/cm^2^ [spectral range]	single result wavelength in nm	single log-reduction dose in mJ/cm^2^	Sample [ref.]
2.8 [211–230 nm]	222	2.8	PBS (liquid) [31]
24.1 [251–270 nm] 31.5**	254	14.9	PBS (liquid) [29]
	254	33.3	PBS (liquid) [31]
	254	31.7	SMG (liquid) [this study]
	254	*5.3 *6.0	gelatin-medium (surface) [33]
	254	*reduction observed but no dose available	steel disks, N95 respirator and plastic bins (surface) [30],[63]
	270	31.3	PBS (liquid) [31]
40 [271–290 nm]	282	40	PBS (liquid) [31]
98 [311–330 nm]	311	980	SMG (liquid) [this study]
14 684 [351–370 nm]	365	14 684	SMG (liquid) [this study]

*Note: Results marked with “*” were not included in the determination of the median, because they were obtained on very different surfaces or information like irradiation dose was missing. “**” median log-reduction dose for Phi6 including the results from this study. (PBS: phosphate buffered saline, SMG: saline magnesium gelatin buffer, both exhibit low UV absorption.)

For the UVB and UVA data there are no published Phi6 log-reduction doses for comparison, but some UV results for the ssRNA bacteriophage MS2, with log-reduction doses of about 7 mJ/cm^2^ at 222 nm, 14–30 mJ/cm^2^ in the spectral interval [251–270 nm], 20–63 mJ/cm^2^ in the interval [271–290 nm], 230–330 mJ/cm^2^ in the UVB and 910 mJ/cm^2^ in the UVA range [64]–[67]. The UVC properties of MS2 seem to be very similar to Phi6 in the UVC spectral range. In the UVB and UVA ranges, there is also a large increase for the MS2 log-reduction dose, but–with the limited available data-it seems to be less distinctive than with Phi6, which might just be caused by different applied irradiation spectra.

Unfortunately, the available Phi6 and SARS-CoV-2 data are still sparse and exhibit a high variability. Nevertheless, at least in the spectral range [251–270 nm] data on the Phi6 and SARS-CoV-2 UV sensitivity from different authors are available that seem to fit to another. The results from the other spectral regions are less well funded, but they give a consistent overall picture as shown in Table 3.

Both viruses can be inactivated by UVC, UVB and UVA irradiation, and the here presented relative UV sensitivities decrease with longer wavelengths. However, though both viruses are enveloped RNA viruses the absolute UV sensitivity or necessary log-reduction doses differ, with Phi6 being much more UV resistant than SARS-CoV-2 for UVC, UVB and UVA. This might be caused by differences in the RNA structure and length. Though protein damage might contribute, the main virus inactivation mechanism seems to be caused by RNA/DNA damage [66],[68]–[70]. ssRNA viruses seem to be more UV sensitive than dsRNA ones and longer RNA is more susceptible to ultraviolet radiation than shorter ones [70]. Both properties lead to the result that the 13.5 kbp dsRNA virus Phi6 exhibits higher log-reduction doses than the 30 kbp ssRNA SARS-CoV-2.

**Table 3.** Median log-reduction doses for Phi6 and SARS-CoV-2 and its ratios for different spectral ranges.

Spectral interval	Phi6 median log-reduction dose in mJ/cm^2^	SARS-CoV-2 median log-reduction dose in mJ/cm^2^	ratio
[211–230 nm]	2.8	1.15	2.4
[251–270 nm]	31.5	1.7	18.5
[271–290 nm]	40	2.3	17.4
[291–310 nm]		10.7	91.6
[311–330 nm]	980		
[351–370 nm]	14 684	2 569	5.7

*Note: UVB results for Phi6 and SARS-Co-V-2 are formerly in two different spectral regions but in fact, they are less than 15 nm apart and therefore compared to another.

This confirms the experimental observation that despite structural similarities Phi6 is less UV sensitive than SARS-CoV-2 and therefore Phi6 is not the best suited surrogate for SARS-CoV-2 in UVC, UVB or UVA applications.
